# Investigation on dynamic characteristics of herringbone planetary gear transmission system with staggered tooth phase

**DOI:** 10.1371/journal.pone.0354177

**Published:** 2026-07-21

**Authors:** Shaoshuai Hou, Haichao Ye, Derong Zhu, Shijie Zhang, Jingjing Li, Ming Xia, Sicheng He

**Affiliations:** 1 School of Intelligent Manufacturing, Luoyang Institute of Science and Technology, Luoyang, China; 2 Henan Key Laboratory of Green Building Materials Manufacturing and Intelligent Equipment, Luoyang Institute of Science and Technology, Luoyang, China; 3 School of Artificial Intelligence, Luoyang Normal University, Luoyang, China; 4 School of Materials Science and Engineering, Luoyang Institute of Science and Technology, Luoyang, China; 5 Department of New Product Research and Development, Zhongqi Changxing (Luoyang) Mechanical and Electrical Equipment Engineering Co., Ltd, Luoyang, China; CINVESTAV IPN: Centro de Investigacion y de Estudios Avanzados del Instituto Politecnico Nacional, MEXICO

## Abstract

Vibration and noise are critical indicators that directly affect the transmission characteristics of planetary gearboxes. Consequently, the suppression of vibration and noise has emerged as a major research focus in this field. This paper investigates the meshing mechanism of herringbone planetary gear systems with staggered tooth phase (STP), and derives the analytical formulation for calculating the time-varying mesh stiffness (TVMS) of such gear configurations. In addition, it examines the correlation between the staggered tooth phase angle (STPA) and the system vibration response, as well as the dynamic effects induced by variations in rotational speed and different types of tooth profile errors. The results demonstrate that the implementation of STP could substantially enhance the vibration characteristics of herringbone planetary gear systems. Furthermore, among various tooth profile errors, the single tooth tangential error (STTE) is identified as the dominant factor governing vibration.

## 1. Introduction

Owing to their remarkable merits including high transmission ratio and exceptional power density, herringbone gear is extensively applied in critical engineering equipment such as wind turbines, aircraft engines and marine propulsion systems. Vibration and noise radiation represent key performance metrics for gear transmission systems and require stringent control. Accordingly, the mitigation and regulation of vibration and noise in these systems have evolved into a vital research field [1–3].

TVMS is a major excitation of transmissions; it was computed by many scholars [4,5]. Dai came up with an upgraded analytical model accounting for modifications made to the addendum part as well as varying loading circumstances so as to figure out how hard the gears meet each other up via contact mechanics plus ideas behind when things come together within a wheel setup [6]. Li introduced an analytical approach formulated from Hertz contact theory, considering free-end effects and contact gap variations, thereby achieving higher computational efficiency and precision [7]. Yang calculated the mesh stiffness with the teeth modifications and ring flexibility considered [8]. Andary proposed an alternative modeling approach for gear mesh excitations within dynamic simulations, he computed the stiffness of each individual meshing contact pair in advance instead of directly employing the global mesh stiffness [9,10]. Hou put forward a full mathematical model for finding helical gear mesh stiffness with structural joint effect included and also the axial component of meshing force [11,12]. Marafona presented a numerical algorithm for designing gear pairs with constant mesh stiffness, while simultaneously optimizing safety factors and transmission efficiency [13]. Rezaei combined analytical formulations of helical gear sets and planetary systems to find the TVMS [14,15]. Raghuwanshi used a laser displacement sensor to measure the deflections of the gear tooth and then calculated the gear mesh stiffness [16,17].

Dai used a combination of analytic computation methods to look at how spur gear pairs reacted non-linearly: they considered things like how much a spot would move when it was being meshed up and what point that meshing happened to be on in its entire loop. Based off pre-calculated static ones, they used a contact model to get dynamic force at mated gear teeth [18]. Donmez set up a torsion model of an EV drivetrain to study high-speed-specific dynamic behavior by modeling gear-mesh interface as an excitation to represent tooth errors and modification [19,20]. Guilbert has done up something like this with a weirdly shaped set of gears and how the very little or not so much wide parts of them work when spinning [21–23]. Zheng presented an enhanced dynamic model that incorporated gears, and investigated the dynamic behavior of the system [24]. Xie formulated a model via lumped-parameter method incorporating real-time mesh stiffness, and experimentally validated the model through comparative testing [25,26]. Zhang developed a torsion vibrational model of railway vehicle's transmission, including effects of the severity of cracks on the dynamic behavior under torque excitations at both driving end and loaded end [27]. Kong built a gear dynamic model which includes flexible gear bodies and shell elements as well as gyroscopic effect, but still keeps the gear foundation and tooth structure in the finite element formulation [28]. Huangfu brought in a new kind of dynamic model for spalled gear pairs which uses actual spalling forms that came from tiring experiments and looking at how they touch during loaded times to figure out their meshing traits [29]. Cohen validated an analytical model using two experimental test rigs, based on qualitative comparisons of root-mean-square (RMS) vibration responses [30].

Existing literature pays close attention to computing mesh stiffness, employing either lumped masses or finite elements for creation dynamic models of transmissions system. The TVMS of herringbone gears with STP is explored. A dynamic model of a herringbone gear planetary gear train with STP through nodal finite element method is created. Also how STP angle and tooth profile error can affect the vibrations and noises caused by the transmission are studied, and it has a brand-new approach in diminishing these two things within the herringbone gear mechanism.

## 2. The model of the herringbone gears with STP

### 2.1. Structural description of the herringbone gears with STP

**Fig 1:**
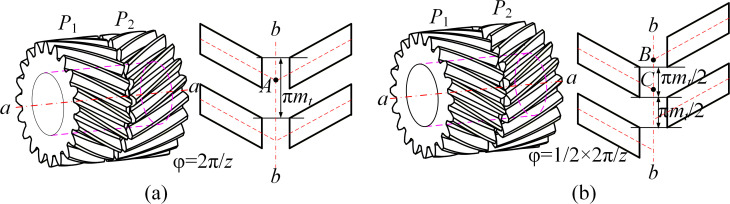
The diagram of herringbone gears with STP.

For standard herringbone gears, left‑handed and right‑handed helical gear have a fixed relationship such that the intersecting points of the extension teeth and center of herringbone symmetry *bb* always coincide, as can be seen at point *A* in Fig 1(a). If the gear *P*_1_ is immobile, and the gear *P*_2_ is being turned round its axis *aa* for an angular position and it could be found that no longer does their intersection line cross with their own gear's symmetry line but rather both gears’ intersection meet up again at places marked out by points *B* & *C* illustrated within Fig 1(b). In operation Gears *P*_1_ inter-engages with its conjugate Gear *P*_2_ inter-engages with its conjugate Gear. These two gear pairs have different meshing moments with each other, and they have a stable phase gap between one and the other. This type of herringbone gear pair is known as the staggered tooth phase (STP). The relative rotational displacement is referred to as the staggered tooth phase angle.

**Fig 2:**
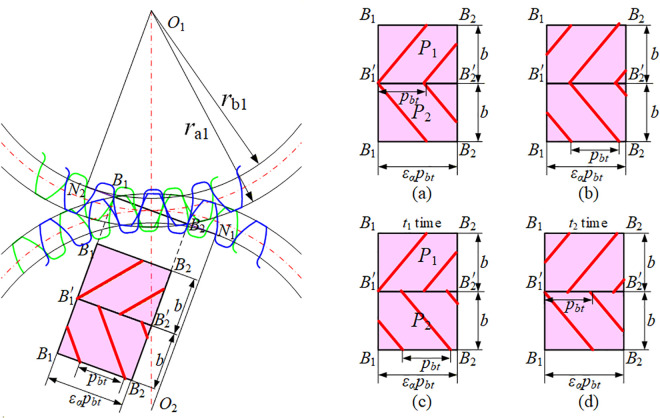
The meshing principle of herringbone gears with STP.

As a herringbone gear pair with STP, the fundamental operating principle is equivalent to the meshing behavior of two independent helical gear pairs. When considering high-speed, precise helical gears with great force, the value of contact ratio for them will usually exceed 2. Taking a meshing pair where the contact ratio ε is between 2 and 3 as an illustration, either dual-tooth meshing or trip-tooth meshing might occur inside the mating region (Fig 2a, 2b).

For the herringbone gear pair with STP, the STP angle is adjustable so that there is a region at *P*_1_ where it has a doubly-tooth engagement state, and there would exist a third tooth engaged between the *P*_2_ pair in such instance. And vice versa when *P*_2_ is engaging in doubly tooth and *P*_3_ is engaging in third tooth (Fig 2c, 2d). Thus, the entire herringbone gear assembly maintains the triple-tooth meshing. This design increases the effective number of simultaneously meshing teeth, thereby enhancing transmission smoothness and reducing vibration and noise.

### 2.2. Model of TVMS of the herringbone with STP

**Fig 3:**
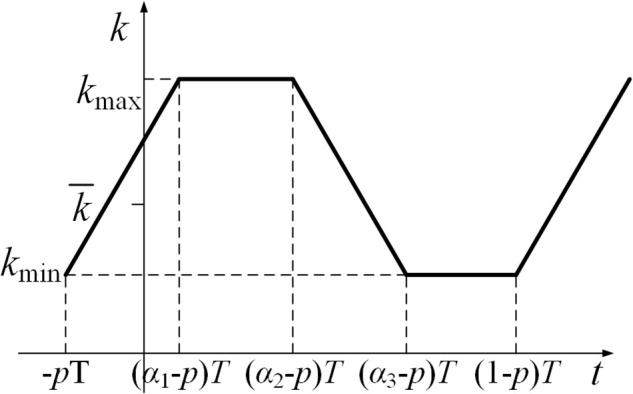
TVMS curve of helical gear.

TVMS of herringbone gear pairs is obtained with respect to two helical gears. In the case where it's only one helical gear pairing, it will be like trapezoid wave form see Fig 3.

Therefore, the mathematics for this curve for a pair of helical gears can be written as:


$$\begin{array}{c} k=\left\{ \begin{array}{c} @l@\frac{k_{max}-k_{min}}{\alpha_{\text{1}}T}\left[t-\left(n-p\right)T\right]+k_{min}nT<t<\left(n+\alpha_{\text{1}}-p\right)T\\ k_{max}\left(n+\alpha_{\text{1}}-p\right)T<t<\left(n+\alpha_{\text{2}}-p\right)T\\ \frac{k_{min}-k_{max}}{\left(\alpha_{\text{3}}-\alpha_{\text{2}}\right)}\left[t-\left(\alpha_{\text{2}}-p+n\right)T\right]+k_{max}\text{\textbackslash{}hspace\{0.5em}\;\}\left(\alpha_{\text{2}}-p+n\right)T<t<\left(\alpha_{\text{3}}-p+n\right)T\\ k_{min}\left(n+\alpha_{\text{3}}-p\right)T<t<\left(n+1-p\right)T\\ \frac{k_{max}-k_{min}}{\alpha_{\text{1}}T}\left[t-\left(n+1-p\right)T\right]+k_{min}\left(n+1-p\right)<t<\left(n+1\right)T \end{array}\right.n=0,1.. \end{array}$$ (1)

*k*_max_ and *k*_min_ represent the maximal and minimal value of helical gear mesh stiffness. *T* is the mesh period. *p* is the engagement position of tooth in gear. It is naturally needed that herringbone gear with STP has *p*_1_ ≠ *p*_2_. And also *α*_1_, *α*_2_, and *α*_3_ are parameters related to contact ratio.


$$\begin{array}{c} \left\{ \begin{array}{c} @l@\alpha_{\text{1}}=min\left({\varepsilon^{\prime }}_{\alpha },1-{\varepsilon^{\prime }}_{\alpha },{\varepsilon^{\prime }}_{\beta },1-{\varepsilon^{\prime }}_{\beta }\right)\\ \alpha_{\text{3}}=\left\{ \begin{array}{c} @l@1-{\varepsilon^{\prime }}_{\gamma }if{\varepsilon^{\prime }}_{\beta }>1-{\varepsilon^{\prime }}_{\alpha }\\ {\varepsilon^{\prime }}_{\gamma }else \end{array}\right.\\ \alpha_{\text{2}}=\alpha_{\text{3}}-\alpha_{\text{1}} \end{array}\right. \end{array}$$ (2)

*ε*_α_, *ε*_β_ and *ε*_γ_ correspond to the transverse, axial, and total contact ratio for the pair of helical gear, respectively. Their fractional parts are expressed as *ε´*_*α*_ = mod(*ε*_*α*_,1), *ε´*_*β*_ = mod(*ε*_*β*_,1), and *ε´*_*γ*_ = mod(*ε*_*γ*_,1).

For simplification of the derivation process, the TVMS *k*(*t*) are rewritten by separating average value and the variable part which is defined as *Δk*(*t*),


$$\begin{array}{c} k\left(t\right)=\overset{―}{k}+\Delta k\left(t\right) \end{array}$$ (3)

The average TVMS is derived based on geometric features of its trapezoidal waveform, with the corresponding mathematical formulation presented below.


$$\begin{array}{c} \overset{―}{k}=\frac{\text{1}}{\text{2}}\left(k_{max}-k_{min}\right)\left(-\alpha_{\text{1}}+\alpha_{\text{2}}+\alpha_{\text{3}}\right)+k_{min} \end{array}$$ (4)

Furthermore, the variation of TVMS can be established as,


$$\begin{array}{c} \Delta k\left(t\right)=\sum_{n=1}^{\infty }\left(a_{n}cos\frac{2n\pi t}{T}+b_{n}sin\frac{2n\pi t}{T}\right) \end{array}$$ (5)

Where,


$$\begin{array}{c} \left\{ \begin{array}{c} @l@a_{n}=\frac{k_{max}-k_{min}}{2{\left(n\pi \right)}^{\text{2}}}\left\{\begin{array}{c} @l@+\frac{\text{1}}{\alpha_{\text{1}}}cos\left[2n\pi \left(\alpha_{\text{1}}-p\right)\right]+\frac{\text{1}}{\alpha_{\text{3}}-\alpha_{\text{2}}}cos\left[2n\pi \left(\alpha_{\text{2}}-p\right)\right]\\ -\frac{\text{1}}{\alpha_{\text{3}}-\alpha_{\text{2}}}cos\left[2n\pi \left(\alpha_{\text{3}}-p\right)\right]-\frac{\text{1}}{\alpha_{\text{1}}}cos\left(2n\pi p\right) \end{array}\right\}\\ b_{n}=\frac{k_{max}-k_{min}}{2{\left(n\pi \right)}^{\text{2}}}\left\{\begin{array}{c} @l@+\frac{\text{1}}{\alpha_{\text{1}}}sin\left[2n\pi \left(\alpha_{\text{1}}-p\right)\right]+\frac{\text{1}}{\alpha_{\text{3}}-\alpha_{\text{2}}}sin\left[2n\pi \left(\alpha_{\text{2}}-p\right)\right]\\ -\frac{\text{1}}{\alpha_{\text{3}}-\alpha_{\text{2}}}sin\left[2n\pi \left(\alpha_{\text{3}}-p\right)\right]+\frac{\text{1}}{\alpha_{\text{1}}}sin\left(2n\pi p\right) \end{array}\right\} \end{array}\right. \end{array}$$ (6)

Average mesh stiffness of the helical gears during one mesh cycle has dependence to the node position *α*_1_, *α*_2_ and *α*_3_. But the mesh stiffness changes with time because both the *α*_1_, *α*_2_ and *α*_3_ as well as the phase angle *p* affect them.

A herringbone gear pair with STP structure consists of two helical gear pairs. The mesh stiffness of the two helical gear pair could be denoted by *k*^(1)^(*t*) and *k*^(2)^(*t*), their initial mesh phases could be denoted by *p*^(1)^ and *p*^(2)^, and the superscripts (1) and (2) are used to represent the two gear pairs. On this basis, the time-varying mesh stiffness of the two helical gear pairs could be mathematically formulated as follows.


$$\begin{array}{c} k^{\left(1\right)}\left(t\right)=\overset{―}{k}+\Delta k^{p\left(1\right)}\left(t\right)k^{\left(2\right)}\left(t\right)=\overset{―}{k}+\Delta k^{p\left(2\right)}\left(t\right) \end{array}$$ (7)

$\overset{―}{k}$ represents the average value of the mesh stiffness of a single-sided meshing pair within one meshing period, and *Δk*^*p*(1)^ and *Δk*^*p*(2)^ represent the variation parts of the mesh stiffness of the two meshing pairs, respectively. Obviously, *p*(1)≠*p*(2), *Δk*^*p*(1)^≠*Δk*^*p*(2)^.

The mesh stiffness of the single-sided meshing pair of the herringbone gear with STP could be obtained by taking the average of the mesh stiffness of the two meshing pairs. Thus, considering the STP, the mesh stiffness of each gear pair could be written as,


$$\begin{array}{c} k_{d}\left(t\right)=\frac{\text{1}}{\text{2}}\left[k^{\left(1\right)}\left(t\right)+k^{\left(2\right)}\left(t\right)\right] \end{array}$$ (8)

Furtherly,


$$\begin{array}{c} k_{d}\left(t\right)=\overset{―}{k}+\frac{\text{1}}{\text{2}}\left[\underset{\Delta k}{\underbrace{\Delta k^{p\left(1\right)}\left(t\right)+\Delta k^{p\left(2\right)}\left(t\right)}}\right] \end{array}$$ (9)

Here, Δ*k* denotes the mesh stiffness fluctuation, which can be further derived via Fourier series expansion.


$$\begin{array}{c} \Delta k=\sum_{n=1}^{\infty }\left\{\sqrt{{\left(a_{n}^{d}\right)}^{\text{2}}+{\left(b_{n}^{d}\right)}^{\text{2}}}sin\left(\frac{2n\pi t}{T}+\varphi \right)\right\} \end{array}$$ (10)

The relevant term ${ℳ}_{rpi}{\ddot{q}}_{rpi}+{𝒞}_{rpi}{\dot{q}}_{rpi}+{𝒦}_{rpi}q_{rpi}={ℱ}_{rpe}+{ℱ}_{rpi}$ represents the initial meshing phase of herringbone gears with STP.

Given that the initial meshing phases *p*^(1)^ and *p*^(2)^ of an STP herringbone gear are inherently different, a reasonable simplification is adopted by setting *p*^(1)^=0 and *p*^(2)^= *p*. Accordingly, for each helical gear meshing pair:


$$\begin{array}{c} \left\{ \begin{array}{c} @l@a_{n}^{d}=-Q_{x}\left\{\frac{sin\left[n\pi \left(\alpha_{\text{1}}-p\right)\right]sin\left(n\pi \alpha_{\text{1}}\right)}{\alpha_{\text{1}}}+\frac{sin\left[n\pi \left(\alpha_{\text{2}}-\alpha_{\text{3}}\right)\right]sin\left[n\pi \left(\alpha_{\text{2}}+\alpha_{\text{3}}-p\right)\right]}{\alpha_{\text{3}}-\alpha_{\text{2}}}\right\}\\ b_{n}^{d}=Q_{x}\left\{\frac{cos\left[n\pi \left(\alpha_{\text{1}}-p\right)\right]sin\left(n\pi \alpha_{\text{1}}\right)}{\alpha_{\text{1}}}+\frac{sin\left[n\pi \left(\alpha_{\text{2}}-\alpha_{\text{3}}\right)\right]cos\left[n\pi \left(\alpha_{\text{2}}+\alpha_{\text{3}}-p\right)\right]}{\alpha_{\text{3}}-\alpha_{\text{2}}}\right\}\\ Q_{x}=\frac{2\left(k_{max}-k_{min}\right)cos\left(n\pi p\right)}{{\left(n\pi \right)}^{\text{2}}} \end{array}\right. \end{array}$$ (11)

Amplitude of the *n*^th^ -order for the mesh stiffness is defined as follows:


$$\begin{array}{c} \sqrt{{\left(a_{n}^{d}\right)}^{\text{2}}+{\left(b_{n}^{d}\right)}^{\text{2}}}=\left|Q_{x}\right|\sqrt{\begin{array}{c} \frac{{sin}^{\text{2}}\left(n\pi \alpha_{\text{1}}\right)}{{\left(\alpha_{\text{1}}\right)}^{\text{2}}}+\frac{{sin}^{\text{2}}\left[n\pi \left(\alpha_{\text{2}}-\alpha_{\text{3}}\right)\right]}{{\left(\alpha_{\text{3}}-\alpha_{\text{2}}\right)}^{\text{2}}}\\ +2\frac{sin\left(n\pi \alpha_{\text{1}}\right)sin\left[n\pi \left(\alpha_{\text{2}}-\alpha_{\text{3}}\right)\right]}{\alpha_{\text{1}}\left(\alpha_{\text{3}}-\alpha_{\text{2}}\right)}cos\left[n\pi \left(\alpha_{\text{1}}-\alpha_{\text{2}}-\alpha_{\text{3}}\right)\right] \end{array}} \end{array}$$ (12)

It could be observed that the amplitude of the variation in mesh stiffness of the gear with STP is related to *α*_1_, *α*_2_, *α*_3_, and *p*. That is to say, the amplitude of the mesh stiffness of the gear with STP is not only related to the amount of the STP angle, but also to the contact ratio of the gear.

**Fig 4:**
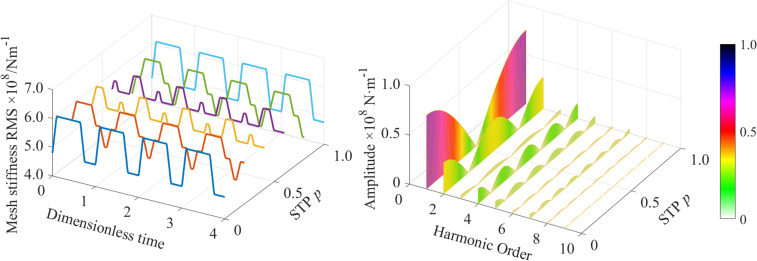
TVMS at different STP angles (a) Time domain representation, and (b) Frequency do-main representation.

TVMS of gears with various STP angle is shown in Fig 4.

Helical gears with STP have less fluctuating mesh stiffness compared to regular gear pairs. In the added frequency domain STP configuration has its own amplitude variations on the related meshes stiff harmonics as well.

## 3. The dynamic model of the herringbone gears with STP

### 3.1. The model of the mesh unit

**Fig 5:**
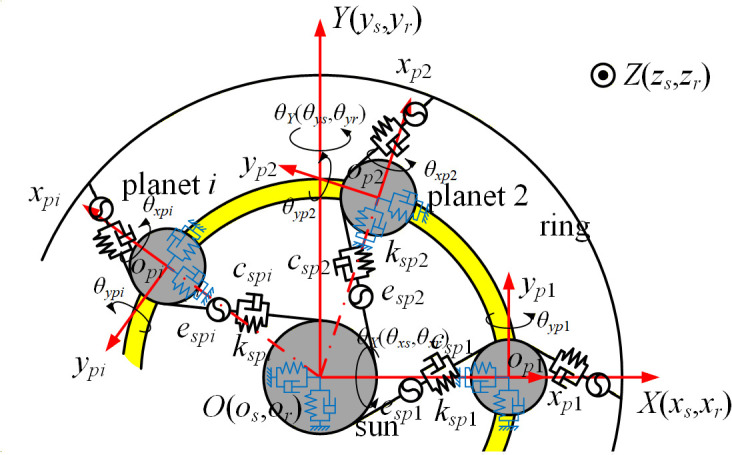
Meshing relationship of the herringbone planetary gear transmission system.

General coordinates could be established, and horizontal line is taken as *x*-axis and vertical axis as *y*. The local coordinate systems for the sun and ring gears will also be based on this. The local coordinate system of planetary gear is defined with the coordinate origin at the center of the planetary gear, the *x*-direction is defined along the vector that connects the sun and planetary gear center point, and the *y*-direction are defined as orthogonal to this vector. The *z* for both driving and driven gears are determined by using right hand rule. At the same time, it is assumed that *φ*_spi_ is the connection line of the connecting line with respect to *x*-axis. And *α*_sp_ is referred to as the meshing angle, as shown in Fig 5.

The external gear system is formed with sun gear and planet gear. Their dynamics equation is controlled by the below formula through Newton’s second law.


$$K_{spi}=k_{spi}V_{spi}^{T}V_{spi}$$ (13)

Where, ***M***_*spi*_=[*m*_*s*_, *m*_*s*_, *m*_*s*_, *I*_*sx*_, *I*_*sy*_, *I*_*sz*_, *m*_*pi*_, *m*_*pi*_, *m*_*pi*_, *I*_*pix*_, *I*_*piy*_, *I*_*piz*_] represent the meshing mass matrix of the external meshing unit. $C_{rpi}=c_{rpi}V_{rpi}^{T}V_{rpi}$, $K_{rpi}=k_{rpi}V_{rpi}^{T}V_{rpi}$ respectively representing the meshing damping and mesh stiffness matrix. $F_{rpe}=C_{rpi}V_{rpi}^{T}e_{rpi}+K_{rpi}V_{rpi}^{T}e_{rpi}$indicate the load excitation caused by error. ***F***_*spi*_ denote external load.

A meshing vector for an external gear set can be seen from ref. [31].

As for dynamic equation, this one is formed from two things: a ring and planet gear working together inside an internal gear set,


$$K_{rpi}=k_{rpi}V_{rpi}^{T}V_{rpi}$$ (14)

Where, ***M***_*rpi*_=[*m*_*pi*_, *m*_*pi*_, *m*_*pi*_, *I*_*pix*_, *I*_*piy*_, *I*_*piz*_, *m*_*r*_, *m*_*r*_, *m*_*r*_, *I*_*rx*_, *I*_*ry*_, *I*_*rz*_] represent the meshing mass matrix of the internal meshing unit.$M_{ij}{\ddot{X}}_{ij}+\left(C_{ij}+\Omega G_{ij}\right){\dot{X}}_{ij}+K_{ij}X_{ij}=\text{0}$, ${ℳ}_{bi}{\ddot{𝒳}}_{bi}+{𝒞}_{bi}{\dot{𝒳}}_{bi}+{𝒦}_{bi}{𝒳}_{bi}=\text{0}$ respectively representing the meshing damping and mesh stiffness matrix of the internalmeshing pair. ${ℳ}_{z}{\ddot{𝒳}}_{z}+\left({𝒞}_{z}+\Omega {𝒢}_{z}\right){\dot{𝒳}}_{z}+{𝒦}_{z}{𝒳}_{z}={ℱ}_{z}+{ℱ}_{e}+{ℱ}_{f}$ indicate the load excitation caused by error. ***F***_*rpi*_ denote external load of the internal mesh pair.

A meshing vector for an internal gear set also can be seen from ref. [31].

### 3.2. The model of the shaft unit

For each discrete shaft segment element, external forces are applied exclusively at its two endpoints, thereby inducing bending, torsional, and axial deformations within the segment. Accordingly, the mechanical behavior of such shaft elements can be analyzed using spatial beam theory. Shaft type component shows bending deformation and lateral displacement, so, the Timoshenko’s beam is used for creating dynamic equation of shaft unit.

**Fig 6:**
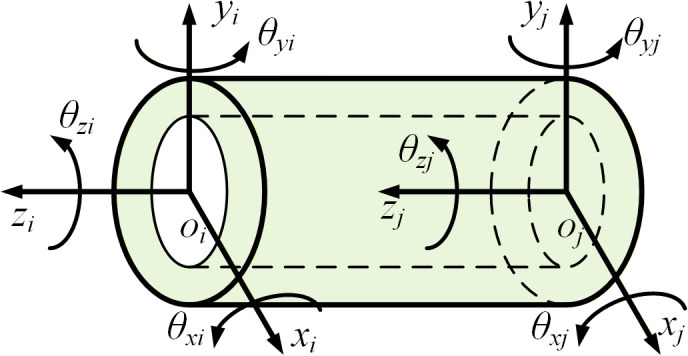
Schematic diagram of two node shaft segment element.

Within the generalized coordinate system *o*-*xyz*, 2-node Timoshenko beam element is defined by translational displacements and rotational angles at its nodes. For nodes *i* and *j*, the displacements are represented as *x*_*i*_, *y*_*i*_, *z*_*i*_ and *x*_*j*_, *y*_*j*_, *z*_*j*_, respectively. Meanwhile, the rotational angles about these three axes are defined as *θ*_*xi*_, *θ*_*yi*_, *θ*_*zi*_ and *θ*_*xj*_, *θ*_*yj*_, *θ*_*zj*_, as illustrated in Fig 6.

The mathematical equation of the shaft unit could be written as,


$$\begin{array}{c} M_{ij}{\ddot{X}}_{ij}+\left(C_{ij}+\Omega G_{ij}\right){\dot{X}}_{ij}+K_{ij}X_{ij}=\text{0} \end{array}$$ (15)

Herein, ***K***_*ij*_, ***M***_*ij*_, and ***G***_*ij*_ represent the stiffness, mass, and gyroscopic matrix of the Timoshenko beam, while ***C***_*ij*_ denotes corresponding damping matrix.

### 3.3. The model of the support unit

**Fig 7:**
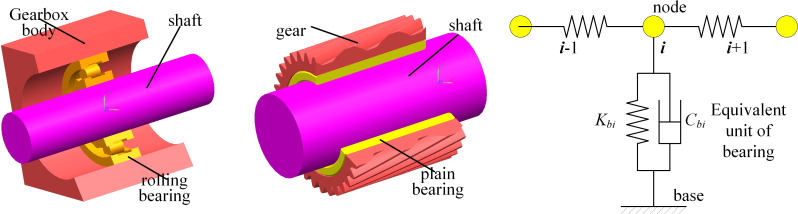
Bearing support structure, (a) rolling bearing, (b) plain bearing, (c) equivalent unit of bearing.

Bearings connect the gearbox housing to the transmission shafts, providing structural support, reducing rotational friction. Each bearing is simplified into an equivalent spring–damping element, as illustrated in Fig 7.

The dynamic equation governing the coupling element between the bearing and gearbox housing can be expressed as follows:


$$\begin{array}{c} M_{bi}{\ddot{X}}_{bi}+C_{bi}{\dot{X}}_{bi}+K_{bi}X_{bi}=\text{0} \end{array}$$ (16)

The displacement vector of bearing node *i* is represented by ***X***_***bi***_, and the associated mass, damping, and stiffness matrices are given as ***M***_***bi***_, ***C***_***bi***_, and ***K***_***bi***_, respectively.

### 3.4. The system-level dynamic model

**Fig 8:**
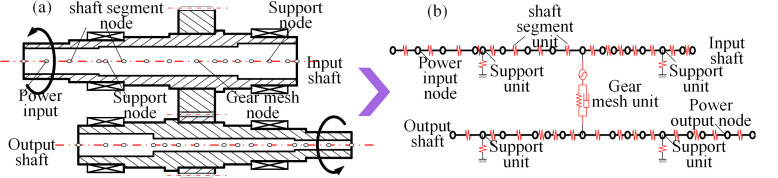
Parallel shaft gear transmission system (a) geometric model diagram (b) Finite Element Discrete Model.

Figure 8 presents the schematic diagram of a simple parallel-shaft transmission system. According to the geometric configuration and spatial layout of the shafts, the key nodes of the system are categorized into gear meshing nodes, shaft segment nodes, bearing support nodes, power input nodes, and load output nodes. Each discrete shaft segment is modeled as a beam element, with approximations adopted for its frequency characteristics and rotational inertia. Adjacent beam elements are interconnected via spring constraints. The gear meshing unit is modeled to behave like a spring damper and coupled with the shaft segment. The bearing support unit is regarded as being made up of a spring support; this connection takes place in line with a particular location along the length of the shaft.

According to above described shaft segments and coupling procedure, a dynamic model of herringbone planetary gear transmission system is developed using a particular type of GTF gear transmission system with given geometry along with its related gear parameters (Table 1).

**Table 1:** The basic parameters.

	Modulus /mm	Tooth width /mm	Pressure angle /°	Helical angle/°	Number of teeth	modification coefficient
Sun gear	3	46	22.5	30	34	0.1016
Planetary gear					31	0.1235
Ring gear					96	0.3485

The model includes the 6 DOF displacements of the left and right sun gears, planetary gears, and ring gear with different kinds of excitation like TVMS, tooth-surface friction and mesh error being taken into consideration. In the model there are five planetary gears that are distributed around in the circle. Left and right gear in model is noted by “l”(left) and “r”(right) respectively.

**Fig 9:**
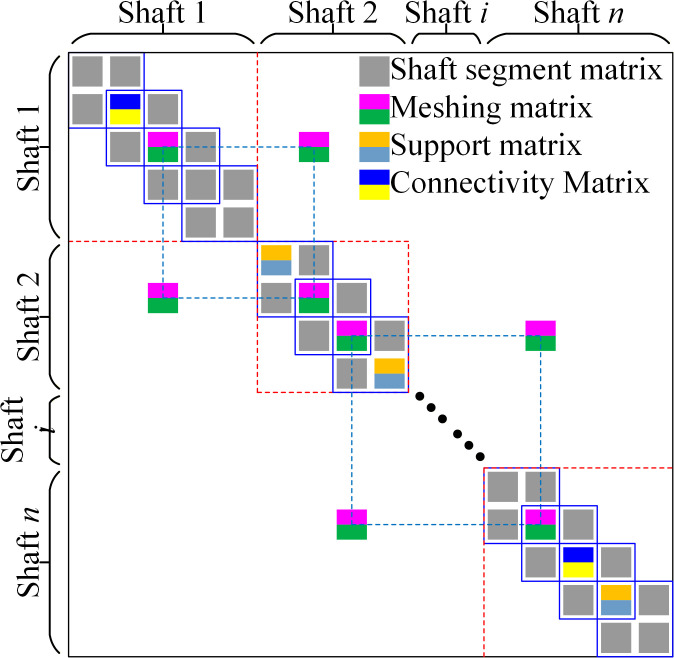
Matrix assembly diagram.

The overall mass, stiffness, damping, and gyroscopic matrices, together with the external load vector, are assembled in accordance with the nodal coordinate displacements, as shown in Fig 9. The two-colored square symbols in the figure indicate the coupling relationships between adjacent nodes.

By assembling the system matrices, the dynamic differential governing equation for the investigated GTF herringbone planetary gear transmission system is derived and formulated as:


$$\begin{array}{c} M_{z}{\ddot{X}}_{z}+\left(C_{z}+\Omega G_{z}\right){\dot{X}}_{z}+K_{z}X_{z}=F_{z}+F_{e}+F_{f} \end{array}$$ (17)

where ***M***_*z*_, ***K***_*z*_, ***C***_*z*_, and ***G***_*z*_ are the integrated mass, stiffness, damping, and gyroscopic matrix. ***F***_*z*_ represents the external load vector (including the input torque and resistive load). ***F***_*f*_ corresponds to tooth friction excitation vector.

## 4. Results and discussion

### 4.1. Basic Parameters

Fundamental Geometric Parameters of certain GTF gear is listed in Table 1.

### 4.2. The influence of STP angle

**Fig 10:**
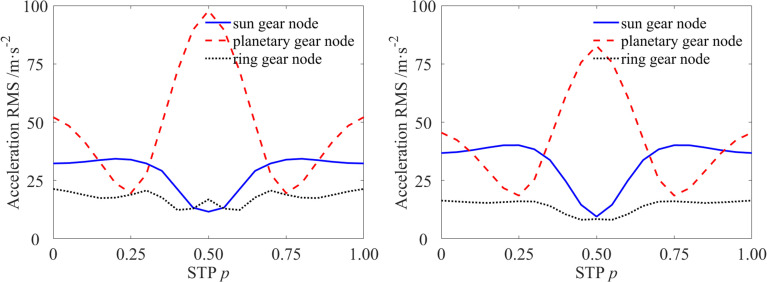
The relationship between acceleration of planetary gear system and STP, (a) Right helical gear node, (b) Left helical gear node.

The vibration responses of key components within the herringbone planetary gear train were investigated under various STP conditions. Figure 10 illustrates the relationships between the vibration acceleration RMS and the STP of the sun, planet and ring gear.

Vibration acceleration curves of the gears exhibit symmetric characteristics with variations in STP parameter, corresponding to a symmetry factor of *p* = 0.5. Minor differences in vibration acceleration exist between the left and right sun gears; the left sun gear presents slightly lower acceleration due to its closer proximity to the power input node. Meanwhile, the left planet gear delivers marginally higher acceleration than its right counterpart.

**Fig 11:**
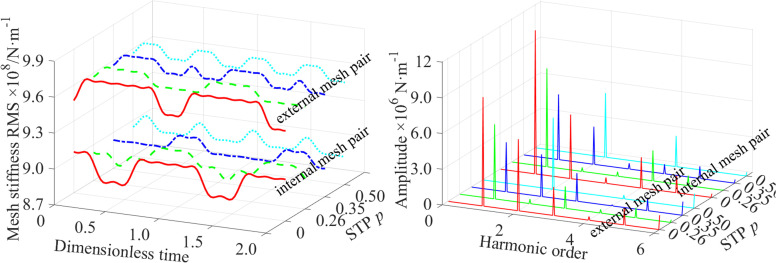
Schematic diagram of external / internal mesh stiffness.

Under different STP configurations, the herringbone planetary gear system achieves symmetric vibration responses on both sides. Accordingly, the result of left gear is selected for detailed analysis. Within the range of *p* = 0 to *p* = 0.5, the planet gear node exhibits the most pronounced variation in vibration acceleration, reaching approximately 78 m/s^2^. In comparison, the corresponding variations for the sun and ring gear are relatively limited, at roughly 22 m/s^2^ and 9 m/s^2^, respectively. As STP increases, vibration acceleration of planet gear initially decreases and subsequently increases, attaining its maximum at *p* = 0.50 and its minimum at *p* = 0.26. This phenomenon occurs because the planet gear is simultaneously excited by both internal and external time‑varying mesh stiffness, with an inherent phase difference existing between them (Fig 11). At *p* = 0.5, the mesh stiffness amplitude becomes maximum at 2 mesh frequency, hence the planetary gear’s vibration acceleration would be maximum too.

According to the research results on one pair herringbone gear with STP, the optimal STP is min(*α*_2_, 1-*α*_2_). Therefore, for a herringbone planetary gear system with STP, the optimal STP is the optimal STP of the external meshing pair *p*_ex_, the internal meshing pair *p*_in_, or some combination of the two. According to the parameters in this article, it could be calculated that, *p*_ex_ = 0.26, *p*_in_ = 0.35. In planetary gear systems, the STP structure has the greatest impact on planetary gear vibration. Therefore, the planetary gear vibration could be taken as the evaluation object. When the planetary gear vibration is at its minimum, the corresponding STP is 0.26. Obviously, this is consistent with the smaller of *p*_ex_ and *p*_in_. Due to the fact that the vibration response of the planetary gear varies with the change of the STP, the vibration response of the planetary gear is minimized when the STP is changed to *p* = min(*p*_ex,_ *p*_in_). Therefore, it is believed that the optimal STP of the herringbone planetary gear system is *p* = min(*p*_ex,_ *p*_in_).

**Fig 12:**
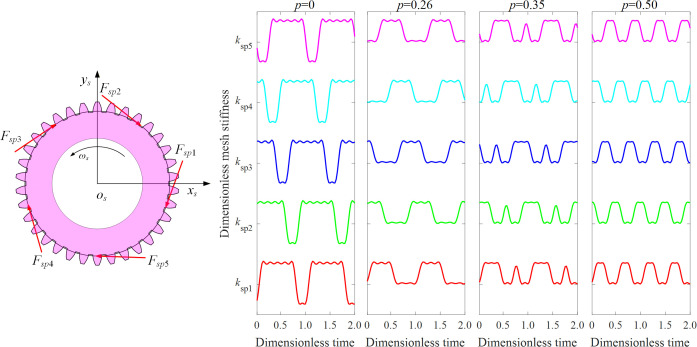
The force of sun gear and external mesh stiffness curve.

When the STP gets bigger, vibration acceleration for central gears remains relatively stable and decreases only slightly near *p* = 0.5. Since the meshing forces are distributed uniformly around the center gear (Fig 12). The single gear pair mesh stiffness usually causes the central gear’s vibration acceleration to first decrease and then increase as the STP is increased, but because many planetary gears engage the central gear at once, the impact of STP on vibration is unimportant. A STP dependent reduction method for the planetary gears is not present in the case of single parallel shaft gear transmission system, this is the main difference between the two types.

**Fig 13:**
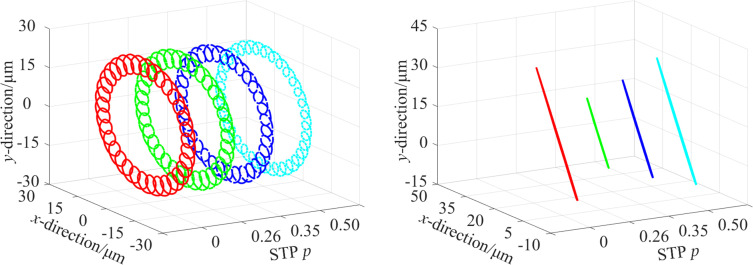
The trajectory of the Planetary gear system, (a) sun gear node, (b) planetary gear node.

The vibration response of the transmission system is further analyzed with four typical STP parameter values: *p* = 0, 0.26, 0.35, and 0.50. The vibration trajectories of each structural component are presented in Fig 13. Due to the similar vibration behaviors of the two central gears, only trajectory curves for sun and planet gears under different STP conditions are displayed.

The trajectory for sun gear shows smallest fluctuation at the STP of *p* = 0.5, while highly similar trajectories are observed at *p* = 0.26 and *p* = 0.35. These results indicate that the STP optimization method exerts a limited influence for improving central gear’s vibration displacement. This insensitivity can be explained by the uniform circumferential force distribution provided by the five evenly arranged meshing planet gears, which suppresses the impact of STP‑induced variations in mesh stiffness. In contrast, the planet gears achieve their minimum vibration displacement at *p* = 0.26, with considerably larger motion trajectories occurring at *p* = 0 and *p* = 0.5. This confirms that a well-designed STP scheme can not only effectively regulate the acceleration characteristics of planet gears but also achieve remarkable optimization in vibration displacement control.

### 4.3. The influence of input speed

**Fig 14:**
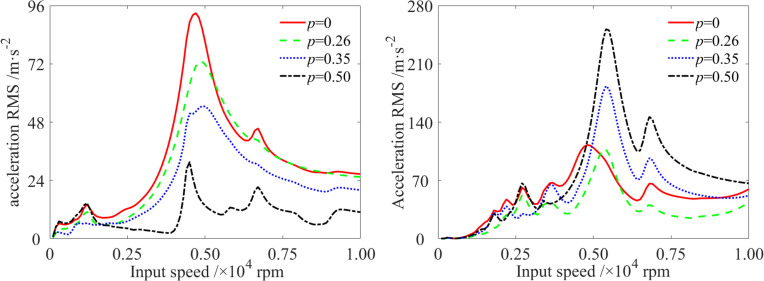
The effect of speed on vibration acceleration at different STP, (a) *x*-direction of sun gear, (b) *x*- direction of planetary gear.

Vibration acceleration of each component in planetary system was analyzed under different rotational speeds, with the results presented in Fig 14. Regarding the central gears, the sun’s vibration data are provided, as the ring gear exhibits nearly identical vibration characteristics and is omitted for conciseness.

The results indicate that both sun’s vibration response and planetary gear increases monotonically with rotational speed, regardless of the STP parameter. Within the speed range from 0 to 10,000 rpm, the sun gear consistently shows lower vibration acceleration than the planet gears. For planet gears, the STP method delivers negligible vibration reduction at low rotational speeds, yet becomes highly effective at high speeds and near resonance points. For the sun gear, the optimal STP parameter of *p* = 0 yields the minimum vibration acceleration; the corresponding results are close to those obtained with *p* = 0.26 and *p* = 0.35, except under resonant conditions. In contrast, the planet gears achieve their lowest vibration acceleration at *p* = 0.26. Furthermore, the adoption of the STP method increases the resonant speed of the planet gears.

**Fig 15:**
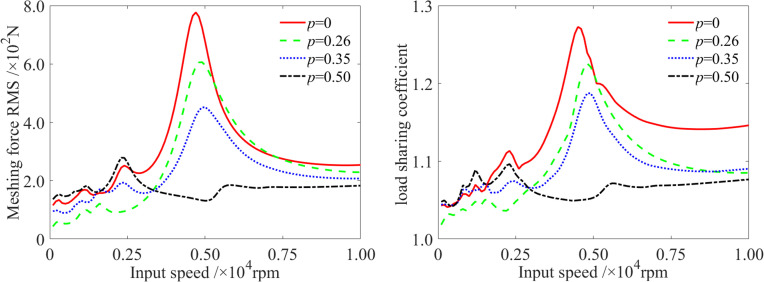
The influence of speed on, (a) c meshing force RMS, (b) load sharing coefficient.

The effect of input speed on meshing force and LSC with various STP conditions is illustrated in Fig 15. It is observed that variations in STP exert little effect on either the dynamic meshing force or the LSC at low speeds. However, a pronounced influence on the LSC emerges at high rotational speeds. Comparative analysis of the curves reveals that a larger STP value corresponds to weaker variation of meshing force and LSC, which aligns with acceleration characteristics of central gear. When STP = 0.5, the resonance point disappears in the dynamic meshing force curve. When STP is set to *p* = 0.5, the resonance peak of meshing force curve disappears. This occurs because the double meshing frequency becomes the dominant excitation frequency, thereby minimizing fluctuations induced by time‑varying mesh stiffness. Additionally, the central gear receives uniformly distributed forces from the five circumferentially arranged planet gears, which further suppresses vibration acceleration and reduces meshing force.

**Fig 16:**
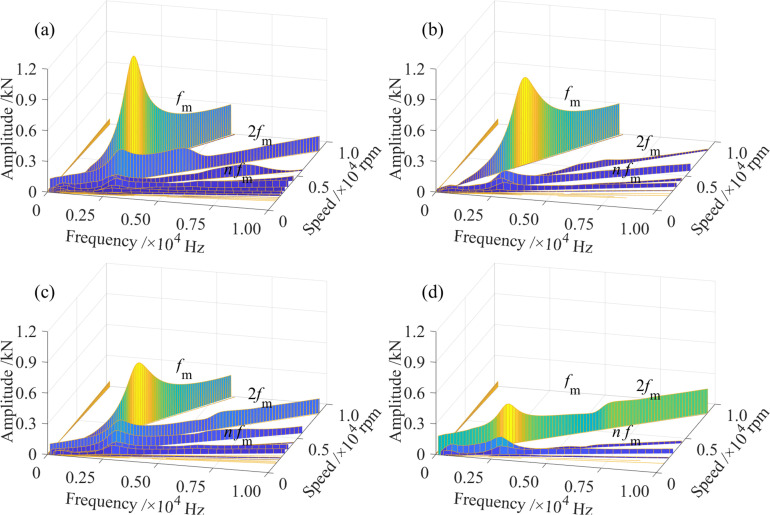
The frequency domain of the external meshing force under different speed: (a) *p* = 0 (b) *p* = 0.26 (c) *p* = 0.35 (d) *p* = 0.50.

The frequency‑domain characteristics of meshing force with variously rotational speeds were investigated, and results are shown in Fig 16.

By comparing the frequency‑domain responses under different STP values, it can be concluded that for *p* = 0, 0.26, and 0.35, the amplitude corresponding to the fundamental meshing frequency initially rises with increasing speed and then remains nearly constant. For the case of *p* = 0.50, the double meshing frequency dominates the excitation, and its amplitude remains essentially unchanged with speed increases.

### 4.4. The influence of the tooth profile error

Slight inherent inaccuracies in manufacturing equipment, along with inevitable deviations arising during machining, will inevitably induce tooth profile errors in gears. Accordingly, exploring how different precision grades affect the dynamic performance. For the studied GTF herringbone gear transmission system, Accuracy Grade 5 is defined as the baseline design; the corresponding tooth profile deviations for gears with alternative accuracy grades are compiled in Table 2.

**Table 2:** Gear geometric error with alternative accuracy grades.

Gear	Cumulative tooth pitch error (CTPE) (μm)			Single tooth tangential error (STTE) (μm)		
	4	5	6	4	5	6
Sun gear	18	25	36	5	7	10
Planetary gear	18	25	36	5	7	10
Ring gear	24	33	47	5.5	8	11

To facilitate comparative analysis of how tooth profile errors impact vibration acceleration across different gear components, relative variation is adopted as the evaluation index. This indicator takes the minimum vibration acceleration obtained under specific tooth profile error conditions as the reference benchmark. The relative variation in acceleration is then calculated based on the deviation between this reference value and the acceleration results measured under other tooth profile error scenarios.

**Fig 17:**
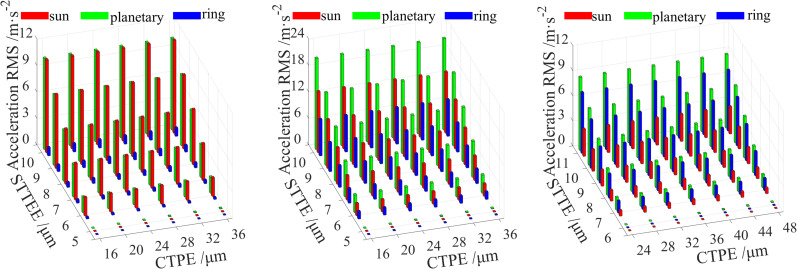
Effect of gear geometric error on vibration acceleration (a) the sun gear geometric error (b) the planet gear geometric error (c) the ring gear geometric error.

This section elaborates on the methodology adopted to quantify effect of sun gear profile errors on vibration acceleration. Vibration acceleration values were calculated under various tooth profile error conditions. The minimum vibration acceleration corresponding to specific error parameters (STTE = 5 μm, CTPE = 16 μm, with the relative acceleration normalized to zero) was defined as the reference baseline. Relative acceleration variations were then determined by subtracting this baseline from the acceleration data acquired under other tooth profile error scenarios, as presented in Fig 17(a). It illustrates the individual effects of sun gear profile errors on vibration of planet and ring gear, where the red, green, and blue curves denote different error conditions. Figures 17(b) and 17(c) further demonstrate the contributions of tooth profile errors from planet and ring gear to relative acceleration variations at their respective gear nodes.

Variations in the sun gear’s tooth profile error serve as the primary factor driving fluctuations in relative vibration of sun and planet gears, yielding maximum variations of 10.31 and 10.48 m·s^−2^. In contrast, ring gear shows relatively low sensitivity, with a maximum acceleration variation of only 0.91 m·s^−2^. Tooth profile errors of planet gears induce peak vibration acceleration variations of 20.60 m·s^−2^, 13.77 m·s^−2^, and 8.20 m·s^−2^. Meanwhile, tooth profile errors of the ring gear lead to maximum variations of 3.21 m·s^−2^, 8.72 m·s^−2^, and 7.24 m·s^−2^ for the three gear components above. A gear profile error affects not only its own vibration acceleration but also that of its meshing counterparts. Overall, planet gears profile errors produce the most substantial changes in relative vibration acceleration. Accordingly, it should be regarded as a critical design parameter in the development of herringbone planetary gear train.

The effects of different profile error of sun, planetary & ring gear on dynamic meshing force pairs in transmission is discussed.

**Fig 18:**
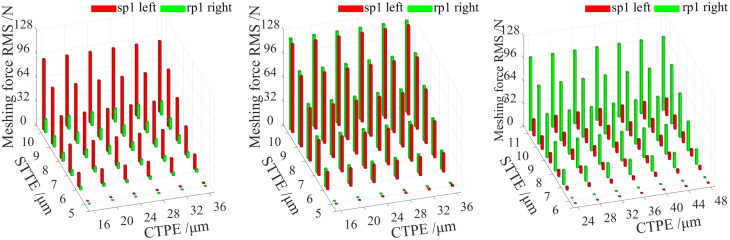
The influence of gear geometric error on dynamic meshing force (a) the sun gear geometric error (b) the planet gear geometric error (c) the ring gear geometric error.

The effect of profile error on vibration acceleration is analogous to that of CTPE, as both exert minimal effects on fluctuations of meshing force. In contrast, STTE is recognized as dominant factor contributing to variations in dynamic meshing force. Specifically, the influence of the profile error of sun gear on meshing force of the external meshing pair is significant, whereas its influence on the internal meshing pair is relatively weak. It’s because that an increase in the planetary gear’s tooth profile error leads to greater equivalent deformation of both meshing pairs, which in turn gives rise to higher dynamic meshing forces. As shown in Fig 18.

Furthermore, the effect of mesh stiffness variations on vibration acceleration and meshing force in transmission system is significantly more prominent than the effect of tooth profile errors on the system’s dynamic response.

## 5. Conclusion

(1) A computational method for obtaining TVMS of herringbone gear with STP is derived, a corresponding dynamic model of herringbone planetary gear system is developed.

(2) The influence of STP on the vibration response of the planetary gear system is investigated. The result indicates the optimal STP value for vibration reduction depends on the specific gear component for which vibration minimization is required. Specifically, an STP value of *p* = 0.50 is identified as the optimal choice for reducing vibration response of central gear. In contrast, to minimize vibration response of planetary gear, the optimal STP value is determined as *p* = min (*p*_w_, *p*_n_), where *p*_w_ and *p*_n_ are the external and internal mesh parameters.

(3) The influence of profile error on vibration response is examined. The findings demonstrate that among all tooth profile error parameters, STTE is the dominant factor contributing to system vibration. Furthermore, the profile error of planetary gear exerts the most significant effect on transmission system.

Due to the limitations of research conditions and time, this study only conducts theoretical analysis, and no experiments are carried out to verify the proposed method and research conclusions. In future work, relevant experimental tests will be implemented to further verify the theoretical research results.

## Supporting information

S1 Data Supplementary data. (XLSX)
